# Supplementary material for: The interactome of intact mitochondria by cross-linking mass spectrometry provides evidence for coexisting respiratory supercomplexes
Source: Mol Cell Proteomics. 2017 Dec 8;17(2):216–32. doi: 10.1074/mcp.RA117.000470 (PMC5795388; doi:10.1074/mcp.RA117.000470)
Supplement: Supplemental Data [file supp_RA117.000470_133922_0_supp_23978_fzffwf.zip › spectra_annotation/mito_spectra_annotation/426-2-1-1-10-1.pdf]

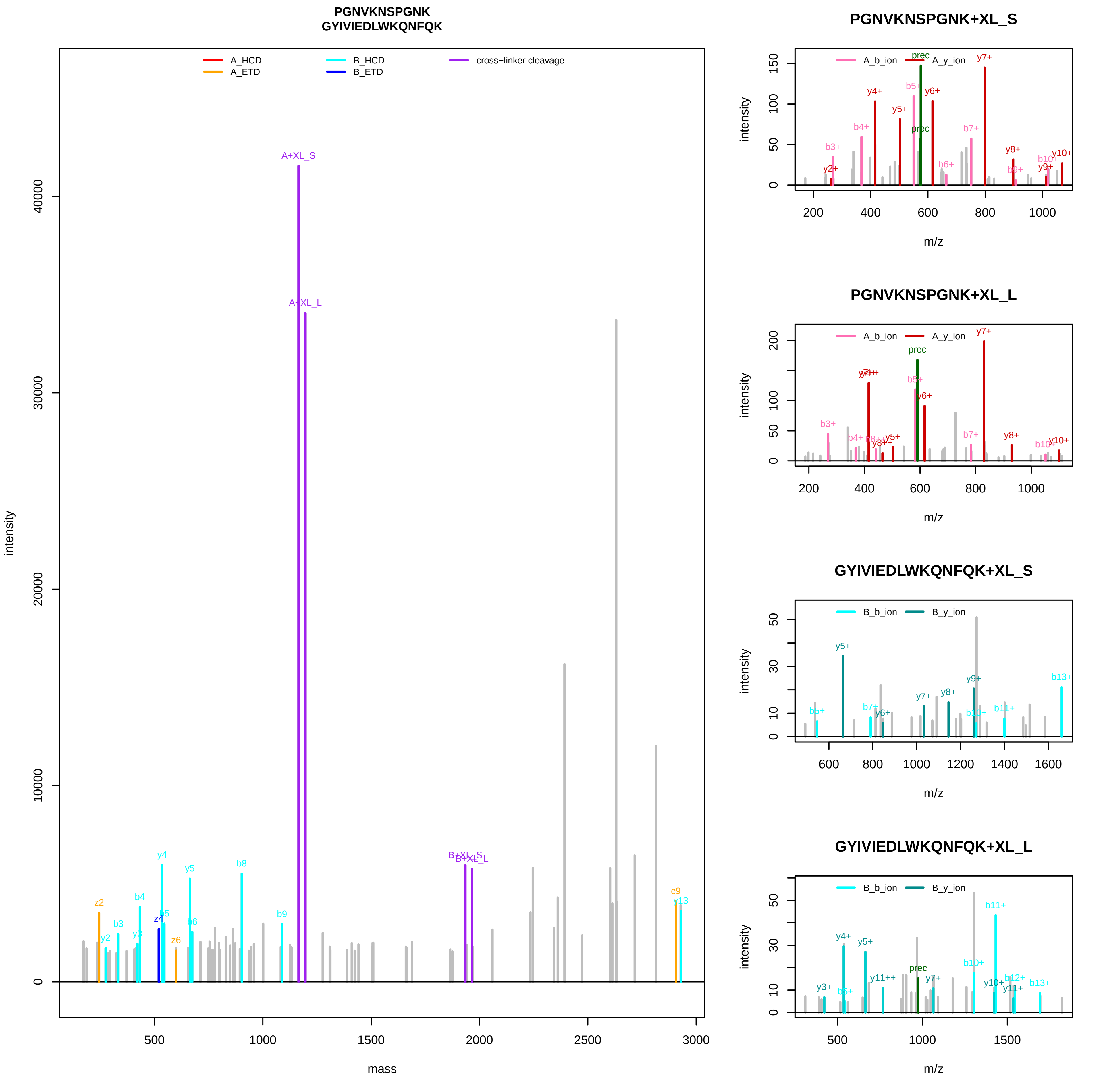
PGNVKNSPGNK
GYIVIEDLWKQNFQK
A_HCD
A_ETD
B_HCD
B_ETD
cross−linker cleavage
A+XL_S
A+XL_L
B+XL_S
B+XL_L
intensity
mass
0
10000
20000
30000
40000
500
1000
1500
2000
2500
3000
z2
y2
b3
b4
z4
y4
b5
z6
y5
b6
b8
b9
c9
y13
PGNVKNSPGNK+XL_S
A_b_ion
A_y_ion
prec
b3+
b4+
b5+
b6+
b7+
b9+
b10+
y2+
y4+
y5+
y6+
y7+
y8+
y9+
y10+
m/z
PGNVKNSPGNK+XL_L
b8++
y8++
GYIVIEDLWKQNFQK+XL_S
B_b_ion
B_y_ion
b5+
b7+
b10+
b11+
b13+
y5+
y6+
y7+
y8+
y9+
GYIVIEDLWKQNFQK+XL_L
y3+
y11++
y10+
y11+
b12+
